# Resorbable scaffolds for alveolar ridge augmentation procedures: A systematic review

**DOI:** 10.1186/s40729-026-00679-1

**Published:** 2026-06-01

**Authors:** Marcus Seiler, Alessandro Cucchi, Markus Tröltzsch, Matthias Tröltzsch, P. W. Kämmerer, Bilal Al-Nawas, Amely Hartmann

**Affiliations:** 1 Clinic for Oral Surgery and Implantology Dr. Seiler und Kollegen MVZ, Filderstadt, Germany; 2 Private Practice, Padua, Italy; 3 Clinic for Oral Surgery and Maxillofacial Surgery, Private Practice Troeltzsch, Ansbach, Germany; 4 Department of Oral and Maxillofacial Surgery, Plastic Surgery, University Medical Centre of the Johannes Gutenberg University of Mainz, Mainz, Germany

**Keywords:** Guided bone regeneration, Resorbable scaffold, Alveolar ridge augmentation, Meshes, Bone augmentation

## Abstract

**Purpose:**

To systematically review the efficacy and safety of resorbable scaffolds for bone augmentation of alveolar bone defects.

**Methods:**

A specific PICO question was formulated: In partially or fully edentulous patients requiring alveolar ridge augmentation (P), how do resorbable scaffolds (I), compared with non-resorbable devices (e.g., titanium meshes) or conventional bone augmentation techniques (C), perform in terms of clinical outcomes including bone gain, implant loss and success, and complication rates (O)? A PubMed/MEDLINE electronic search was conducted to identify clinical studies published until December 2025. No restrictions on publication date were imposed, and only studies published in the English language were considered. Selected studies were all studies on humans using resorbable devices for alveolar ridge augmentation; in vivo and in vitro studies were excluded. Study characteristics, operative techniques, materials, and clinical outcomes including complications, bone augmentation techniques, implant stability, survival and success rates were extracted and analyzed. Resorbable materials evaluated included polylactide (PLA), polycaprolactone (PCL), Polylactic Acid-Polyglycolic Acid (PLGA), and beta-tricalcium phosphate (β-TCP).

**Results:**

A total of 3704 articles were found, but only 7 studies met the pre-established inclusion criteria and were considered suitable for analysis. These studies included 39 patients and 45 edentulous ridges. Implant loss was only reported in one study. Complications occurred in three studies, with graft loss in 5 cases. Primary implant stability values averaged 35Ncm. Degradation time is an important determinant, but remains insufficiently studied/evaluated in the included evidence base.

**Conclusions:**

Resorbable scaffolds seem to represent viable alternatives to conventional bone grafting approache. Customized scaffolds may offer additional esthetic advantages. However, larger randomized controlled trials with longer follow-up periods and standardized outcome measures—including CBCT-based volumetric and linear bone gain, clearly defined complication categories, and implant-related outcomes such as stability, survival, and success—are required to enable robust evidence-based clinical recommendations.

## Background

Successful dental implant osseointegration depends on an adequate and stable bone volume to ensure long-term survival [1, 2]. However, insufficient alveolar bone—stemming from trauma, post-extraction atrophy, or periodontal disease—remains a frequent clinical challenge and often necessitates bone augmentation for optimal implant positioning [3]. Guided Bone Regeneration (GBR) has emerged as one of the most established and well-validated augmentation techniques in contemporary practice [4, 5].

A recent systematic review [6] on peri-implant bone loss following vertical bone augmentation identified guided bone regeneration (GBR) as the most extensively investigated technique and reported favorable outcomes across different follow-up periods, with mean peri-implant bone loss of 0.76 mm in the short term, 1.12 mm in the medium term, and 1.11 mm in the long term.

Central to GBR protocols is the use of barrier membranes, which function as physical barriers to prevent soft tissue infiltration into the augmented site and thereby facilitate bone regeneration [7]. In cases of severe, three-dimensional alveolar bone deficiencies, non-resorbable membranes—such as expanded polytetrafluoroethylene (ePTFE) [8] and dense polytetrafluoroethylene (dPTFE) [9, 10] or customized titanium meshes [11, 12]—have demonstrated superior structural stability and predictable outcomes for successful bone regeneration due to their space-making effect [13, 14].

Despite their high performance in regenerating adequate bone volume, non-resorbable barriers present clinical limitations [15]. Most notably, they require a second surgical intervention for removal with increase of operating time and costs [16]. This additional procedure increases patient morbidity and surgical burden, particularly when unintended osseointegration of the mesh occurs at the crestal margins of the augmented area [17]. Furthermore, soft tissue management around non-resorbable materials can be technically challenging, and exposure or infection during healing represents a well-documented complication [18].

Resorbable meshes offer a compelling alternative by obviating the need for a second surgical procedure and potentially a lower risk of infection [19]. These materials may provide adequate space maintenance, stabilize graft material, and undergo gradual physiologic degradation. A potential challenge relates to the precise control of degradation kinetics, which is hypothesized to align with the timeline of bone healing [20]. Various biocompatible materials have been explored in the literature to address these concerns and optimize clinical outcomes [21].

This systematic review aimed to evaluate the current evidence regarding resorbable scaffolds used for alveolar ridge augmentation in implant dentistry. The primary objective was to describe the efficacy and safety of resorbable devices, while the secondary objective was to identify material characteristics and long-term outcomes.

## Material and methods

### Protocol and registration

The present systematic review´s protocol was registered on PROSPERO (CRD420250650843) and conducted according to the Preferred Reporting Items for Systematic Reviews and Meta-Analysis for Protocols (PRISMA-P 2020) [22].

### Focus question

The systematic search was guided by the following focus question: “ In partially or fully edentulous patients requiring alveolar ridge augmentation (P), how do resorbable scaffolds (I), compared with non-resorbable devices (e.g., titanium meshes) or conventional bone augmentation techniques (C), perform in terms of clinical outcomes including bone gain, implant survival and success, and complication rates (O)?” The question was structured according to PICO criteria.

(P) Population: totally or partially edentulous patients with alveolar ridge deficiencies in need of bone regeneration to place implants. They had to be systemically healthy. Smokers and non-smokers were included.

(I) Interventions: vertical and/or horizontal alveolar ridge augmentation procedures using resorbable scaffolds/barriers/devices. “Resorbable scaffolds” were defined in this review as biodegradable, three-dimensional biomaterial constructs intended to support guided bone regeneration by providing temporary structural support and facilitating tissue ingrowth, while being gradually degraded and replaced by newly formed bone. Osteoconductive scaffold matrices (block-type scaffolds) in terms of customized bone grafts were excluded.

(C) Comparison: alternative surgical procedures with non-resorbable devices.

(O) Outcomes: primary outcome was defined as the vertical and/or horizontal bone gain; secondary outcomes were complication rates (early resorption, exposure, infection, graft loss), opportunity to place implants in the grafted bone site, and survival or success rates.

### Search strategy

A comprehensive electronic literature search was conducted across the following bibliographic databases: PubMed/MEDLINE, the Cochrane Library, and Web of Science (WoS). In addition, a supplementary grey literature search was conducted using Google Scholar to identify potentially relevant studies not indexed in the primary databases. The search was performed on 06 Mar 2025, and updated on 22 Nov 2025. The first 200 results for each search query were screened based on title and abstract. This approach was used as an exploratory supplement to the primary database search and its limitations regarding reproducibility and potential selection bias are acknowledged. No restrictions on publication date were imposed, and only studies published in the English language were considered. The search strategy was developed using a combination of controlled vocabulary terms derived from each database’s thesaurus (e.g., MeSH terms in PubMed) and free-text keywords, informed by previously published literature and refined collaboratively by AH and MT/MT. An initial pilot search was conducted on 8 Oct2024 to test and optimize the search strategy and database-specific filters. Following critical reevaluation and refinement of the search terms and filters, a structured and finalized search was executed on 22 Jan 2025. To ensure completeness and capture newly published studies, the search was subsequently updated on 30 Sept 2025.

**Fig. 1:**
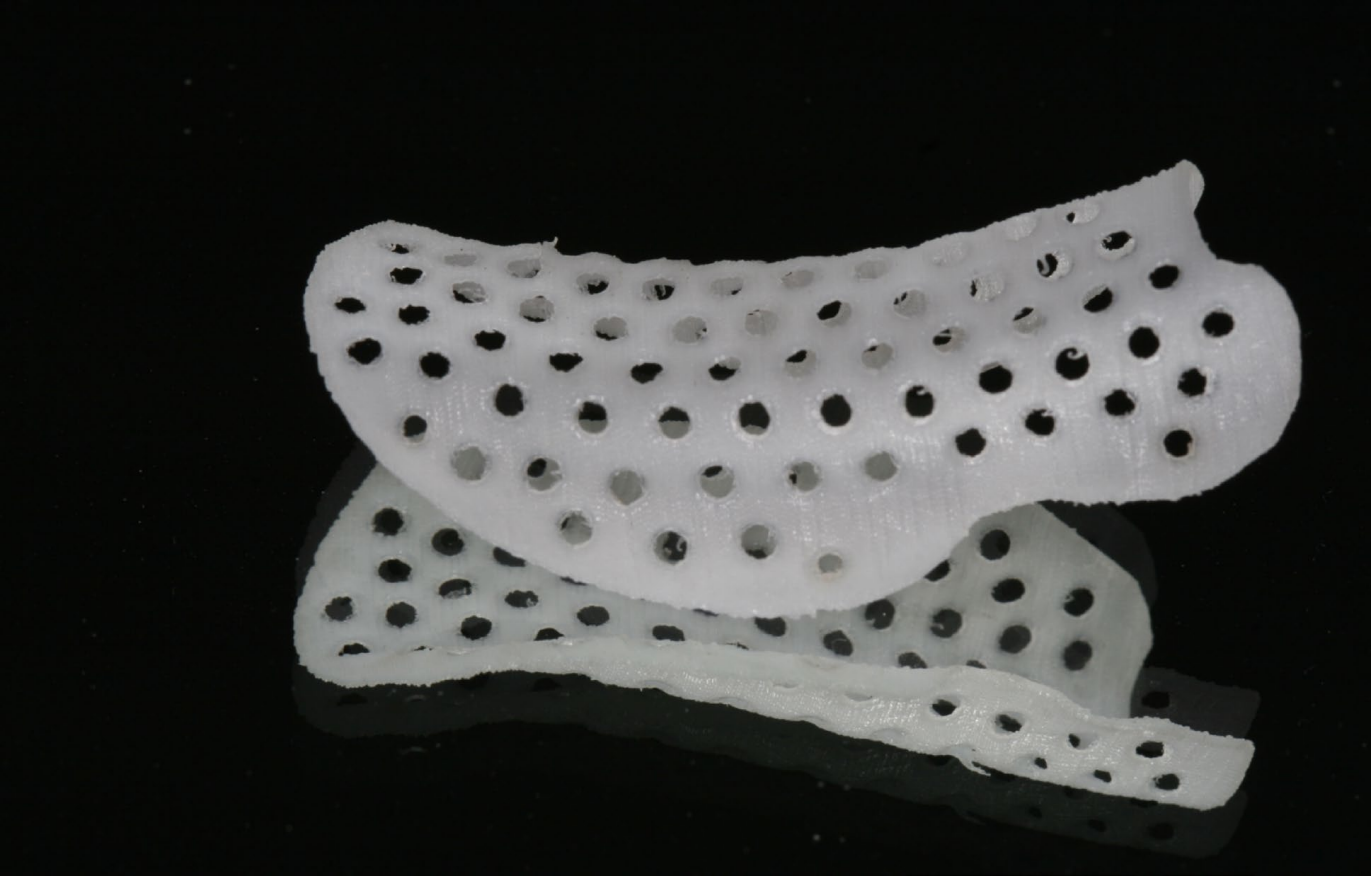
Resorbable scaffold (PLC)

For a comprehensive analysis of these innovative devices, all kinds of clinical studies on humans (randomized controlled clinical trials, prospective studies, cohort studies, case—control studies, cross-sectional studies, case-series, and case reports) were analyzed (Fig. 1).

No restrictions were applied with respect to the year of publication. Preclinical investigations, including both in vitro and in vivo animal studies, were excluded. In addition, non-original research articles—such as letters to the editor, expert opinions, narrative commentaries, conference abstracts, and proceedings without full peer-reviewed manuscripts—were not considered. Studies reporting pooled or aggregated outcomes that did not allow extraction of results specific to the intervention of interest were also excluded. Finally, studies exclusively evaluating non-resorbable scaffolds or devices were excluded from the present review.

The following search terms were used: ("surgical mesh"[MeSH Terms] OR "absorbable implants"[MeSH Terms] OR "biocompatible materials"[MeSH Terms] OR “polycaprolactone” [MeSH Terms] OR "calcium phosphates"[MeSH Terms] OR “polylactide” [MeSH Terms] OR “Polylactic Acid-Polyglycolic Acid Copolymer” [MeSH Terms] OR "surgical mesh"[Title/Abstract] OR "resorbable polymer"[Title/Abstract] OR "resorbable device"[Title/Abstract] OR "resorbable scaffold"[Title/Abstract] OR "resorbable mesh*"[Title/Abstract]) AND ("bone regeneration"[MeSH Terms] OR "bone transplantation"[MeSH Terms] OR "alveolar ridge augmentation"[MeSH Terms] OR "ridge augmentat*"[Title/Abstract] OR "guided bone regenerat*"[Title/Abstract] OR "bone augmentat*"[Title/Abstract] OR "bone reconstruct*"[Title/Abstract] OR "bone transplantation"[Title/Abstract]). Unique search strings were calculated for each database, using the key concepts in different combinations like “Cochrane RCT Sensitivity maximising filter for Pubmed (Revision 2023)”[23]. Additional records were identified through a manual search. Given the low level of available evidence, case series and case reports were explicitly included.

All identified literature was managed with EndNote X9 software by Thomson Reuters. Duplicates were removed electronically and manually.

### Study selection and data extraction

Two independent reviewers (M.S. and A.H.) primarily screened the selected articles at the title and abstract level. Disagreements were solved by discussion and consensus. Any unclarity was resolved by a third author (P.W.K.). Full-text evaluation was independently conducted by the two reviewers. Extracted data and synthesis of the information from the included studies were summarized in three tables reporting publications details like study characteristics (Table 1), materials and methods (Table 2) and study outcome (Table 3).

**Table 1:** Summary of contents according to the study characteristics

Study n	Authors	Year	Country	Study design	Journal	Control group	Sample size	Age	Gender	Number of sites	Jaw of interest	Size of defect	Type of defect	Resorb material
				*RCT, CCT, Pros, Retr, case series, case report*		*Yes or no*	*Number of patients*	*Mean*	*Male / Female*	*Number of treated sites*	*Maxilla / mandible*		*Horizontal or vertical*	*Material*
1	Bartols et al	2018	Germany	Randomzed study	Clin oral implants Res	Yes	30	47.3 years	16 males; 14 females	30	Maxilla (n = 30)	1-tooth (n = 30)	Horizontal	PDLLA
2	Ogata et al	2022	Japan	Case series	J Dent sci	No	5	49.0 years	3 males; 2 females	5	Maxilla (n = 3); mandible (n = 2)	1-tooth (n = 1); 3-teeth (n = 2); 4-teeth (n = 2)	Horizontal	P(LA/CL)
3	Shido et al	2023	Japan	Prospective study	J Clin med	Yes	20	60.8 years	n.s	25	n.s	1-tooth (n = 15); 2-teeth (n = 5);	Horizontal	P(LA/CL)
4	Schonegg et al	2024	Swizterland	Case report	Int J implant dent	No	1	63 years	1 female	1	Mandible (n = 2)	1-tooth (n = 1)	Horizontal	β-TCP
5	Ivanoski et al	2024	Australia	Case report	Clin oral implants Res	No	1	46 years	1 male	1	Maxilla (n = 1)	1-tooth (n = 1)	Horizontal	PCL
6	Takahara et al	2025	Japan	Case series	J oral implantol	No	4	34.5 years	4 males	7	Maxilla (n = 4)	1-tooth (n = 2); 2-teeth (n = 4); 3-teeth (n = 1)	Combined	PLGA
7	Hsu et al	2025	Taiwan	Case series	Clin adv periodontics	No	3	38 years	3 females	3	Mandible (n = 3)	1-tooth (n = 2); 2-teeth (n = 1);	Horizontal	PCL

**Table 2:** Summary of contents according to the intervention characteristics

Study n	Authors	Year	Number of sites	Procedure (intervention)	Material devices	Custom made	Fixation system	Grafting material	membrane	Implant placement	additive	suture	medication	Healing time
			*Number ofTreated sites*	*GBR or khoury or block or others*	*PDLLA, b-TCP, PCL,* *PLGA, PLA,.*		*Tacks or screws*	*DBBM, allograft, autogenous*	*Yes or no*		*PRF, PRGF, BMP,…*	*nylon, PGA, PTFE,.*	*Type of antibiotics e dosage*	*Months before re-entry*
1	Bartols et al	2018	30	SBBT	Poly‐D‐L‐Lactide (test) Autogenous (ctrl)	No	PDLLA tacks	Autogenous + DBBM (test) Autogenous (ctrl)	Native collagen membrane	Delayed (n = 10) (test) Delayed (n = 14) (ctrl)	No	Polyamide	Amoxicillin 750 mg × 3 × 3	4 months
2	Ogata et al	2022	5	GBR	Poly-(L-Lactid Acid / ε-Caprolactone)	No	Titanium tacks	Autogenous + Carbonate Apatite	No	Simultaneous (n = 11)	No	n.s	Amoxicillin 750 mg × 1 × 5	5 months
3	Shido et al	2023	20	GBR	Poly-(L-Lactid Acid / ε-Caprolactone) (test) Native collagen membrane (ctrl)	No	Titanium tacks	Autogenous + Carbonate Apatite	No	Simultaneous (n = 14) Delayed (n = 6)	No	n.s	n.s	5 months (simultaneous) 6 months (delayed)
4	Schonegg et al	2024	1	SBBT	β-Tricalcium phosphate	Yes	Titanium screws	Autogenous	Pericardium membrane	Delayed (n = 1)	No	n.s	n.s	9 months
5	Ivanoski et al	2024	1	GBR	Poly-caprolactone	Yes	Titanium screws	Autogenous + DBBM	Native collagen membrane	Delayed (n = 1)	iPRF	Nylon	Amoxicillin 500 mg × 1 × 5	6 months
6	Takahara et al	2025	4	GBR	Poly-(lactic-co-glycolic acid)	No	PLGA screws	Autogenous + DBBM	No	Delayed (n = 9)	No	Nylon	Amoxicillin 250 mg × 3 × 6	8.5 months
7	Hsu et al	2025	3	SBBT	Poly-caprolactone	No	Titanium screws	Autogenous	No	Simultaneous (n = 4)	No	Nylon	Amoxicillin + Clav.Ac. 1 g × 2 × 7	4 months

**Table 3:** Summary of contents according to the outcomes characteristics

Study n	Authors	Year	Number of sites	Bone gain	Bone volume	Early complication	Late complication	Graft loss	Histo	Remaining material	Implant stability	Osseo- Integration rate	Marginal bone loss	Follow-up of loading	Esthetics
			*Number ofTreated sites*	*Horizontal and/or vertical bone gain*		*Exposure, infection, graft loss, or no*	*Exposure, infection, graft loss, or no*		*Yes or no*	*Yes or no*		*Yes or no*	*Mean* + *st.dv* *(mm)*	*Months*	*Score and values*
1	Bartols et al	2018	15 (test) 15 (ctrl)	4.6 mm (test) 5.1 mm (ctrl)	n.s	33% (n = 5 dehiscence) (test) 7% (n = 1 Infection) (ctrl)	0% (test) 0% (ctrl)	33% (test) 7% (ctrl)	No	n.s	n.s	80% (test) 93% (ctrl)	0.12 mm (test) 0.00 mm (ctrl)	12 months	n.s
2	Ogata et al	2022	5	2.5 mm	n.s	20% (n = 1 infection/fistula)	0%	n.s	No	n.s	n.s	100%	n.s	n.s	n.s
3	Shido et al	2023	10 (test) 10 (ctrl)	2.6 mm (test) 2.3 mm (ctrl)	n.s	0% (test) 0% (ctrl)	0% (test) 0% (ctrl)	n.s	yes	n.s	n.s	n.s	n.s	n.s	n.s
4	Schonegg et al	2024	1	n.s	n.s	No	No	No	Yes	Yes	n.s	100%	n.s	12 months	n.s
5	Ivanoski et al	2024	1	5.5 mm	28.4 mm^3^	No	No	No	Yes	Yes	35 Ncm	100%	n.s	n.s	pes = 7 wes = 9
6	Takahara et al	2025	7	3.1 mm (horiz. gain) 1.7 mm (vert. gain)	n.s	14.3% (n = 1 exposure)	No	n.s	No	n.s	n.s	100%	n.s	n.s	n.s
7	Hsu et al	2025	3	2.1 mm	n.s	No	No	No	No	n.s	35 Ncm	100%	n.s	12 months	n.s

### Data syntheses and analysis

Data extraction was performed manually by different reviewers (A.H., A.C. and M.T./M.T.):

*Study information:* publication details (author, year of publication), country, study design, sample size, control group,

*Patient information:* age (years), sex (male/female), number of sites, jaw of interest (mandible/maxilla), size of defect.

*Intervention information:* procedures (material, devices), customization, fixation, grafting material, membrane, additional material (e.g. Platelet-rich fibrin (PRF), suture, medication, healing time (months).

*Outcomes information:* bone gain (mm), bone volume (mm^3^), early complications, late complications, histology, remaining resorbable material, implant stability, osseointegration, follow-up (months), aesthetics (soft tissue),

### Risk of bias assessment

Risk of bias was assessed at the study level using tools appropriate to the respective study designs. Randomized controlled trials were evaluated using the revised Cochrane Risk of Bias tool according to Higgins and Altman [24]. Non-randomized comparative clinical studies were assessed using the Newcastle–Ottawa Scale (NOS) [25], which evaluates methodological quality across three domains: selection of study groups, comparability of cohorts, and ascertainment of outcomes. Case series and case reports were assessed using the Joanna Briggs Institute (JBI) critical appraisal tools [26]. Risk of bias assessment was performed independently by three reviewers (AH, MS, AC), and any disagreements were resolved by consensus.

## Results

### Study selection

The search strategy identified 3704 records across three databases. After removal of 999 duplicates, 2705 records were screened based on title and abstract, of which 2515 were excluded. A total of 190 records were assessed in more detail, and 39 full-text articles were evaluated for eligibility. Of these, 35 were excluded due to inappropriate study design, outcomes, or publication type (e.g., reviews, book chapters, and meta-analyses).

In addition, a manual search identified 42 records. Of these, 12 were sought for retrieval and 5 were assessed for eligibility, with 2 articles excluded due to irrelevant outcomes (Only in vitro outcomes assessed, focus on material properties without clinical evaluation).

**Fig. 2:**
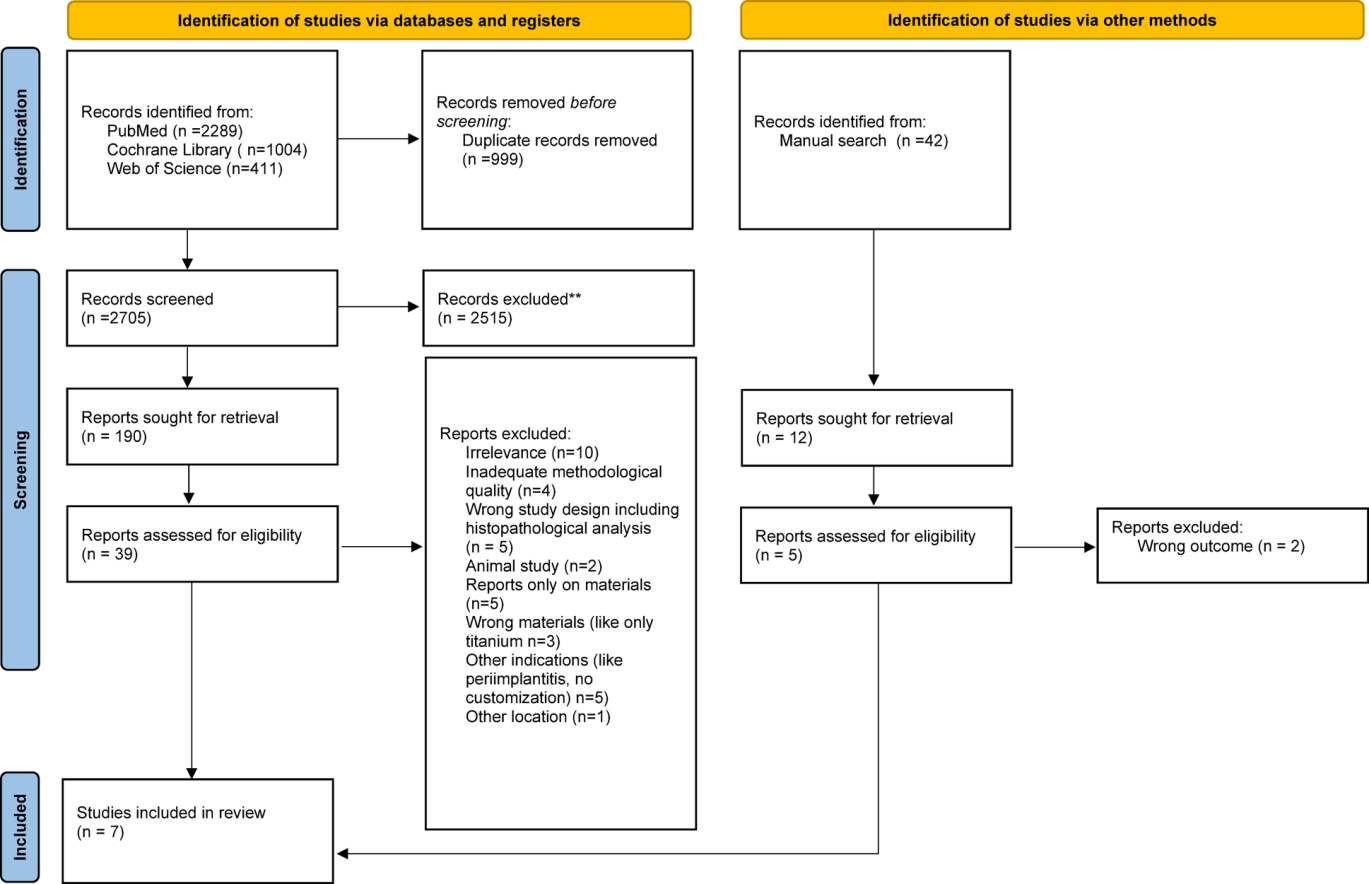
PRISMA 2020—style flow diagram

In total, 7 studies met the inclusion criteria and were included in the review (see PRISMA 2020 flow diagram, Fig. 2).

### Study characteristics

The characteristics of the studies were described in Table 1, reporting the following items: publication details (author, year of publication), country, study design, sample size, control group, age (years), sex (male/female), number of sites, jaw of interest (mandible/maxilla), size of defect.

A total of 7 studies were included. All articles were published in English and represented a wide geographical distribution, spanning 5 countries. The majority of studies were conducted in Japan (n = 3) and one study each from Australia, Germany, Switzerland and Taiwan. All studies employed resorbable scaffolds (customized and non-customized) in complex defects (horizontal/vertical/combined—at least ≥ 3 mm defect size). Except for one randomized controlled clinical trial by Bartols et al. [27], all included studies were clinical prospective and retrospective studies (case reports/series) [28–33]. It should be noted that the total number of participants and sites reported in Table 1 reflects the overall study populations. However, not all cases were treated with the specific intervention of interest or presented with the defect type relevant to this review. Across the included studies, a total of 39 participants and 45 sites met the eligibility criteria and were relevant to the intervention and defect type of interest. Sample sizes were generally small; details per-study (n, male:female where reported) are provided in Table 1.

The duration of follow-up varied across studies and was inconsistently reported. Schönegg et al. [30] reported the longest follow-up of 48 months, whereas Takahara et al. [32] reported a follow-up period of 6–7 months. Ogata et al. [28] and Ivanovski et al. [31] did not report follow-up duration. Moreover, the reference point for follow-up (e.g., post-augmentation or post-implant placement) was not consistently specified.

Included interventions were: (i) bone augmentation procedures using resorbable PLA-based materials [29]; (ii) patient-specific β-TCP scaffolds applied in pre-implant bone grafting [30]; (iii) bone grafting procedures using poly(lactide-co-caprolactone) (P(LA/CL)) [28]; (iv) PCL scaffolds [31, 33]; (v) grafting with resorbable PLGA materials [32]; and (vi) grafting with resorbable poly(L-lactide) devices [27].

### Intervention characteristics

The characteristics of the interventions were described in Table 2, reporting the following items: publication details (author, year of publication), number of sites, jaw of interest (mandible/maxilla), procedures (material, devices), customization, fixation, grafting material, membrane, additional material (e.g. PRF), suture, medication, healing time (months).

The complex defect types ranged from single-tooth defects to larger defects exhibiting both vertical and horizontal bone loss, including type 4 defects according to the Benic et al. classification [6]. One study reported lateral ridge defects [27], further contributing to the heterogeneity of the treated conditions. Although described as complex, some studies placed implants simultaneously with bone augmentation procedure [33]. Treated areas varied from maxillary anterior region [27, 28, 31, 32] in some cases to molar, mandible region like described in Ogata et al. [28] and in Schönegg et al. [30]. Materials of the resorbable scaffolds were Polycaprolactone [31, 33], Poly(lactide-co-glycolid) [28], Poly-D-Lactide [27], β-TCP Beta-tricalcium phosphate[30], Poly(lactic-co-caprolactone) [29], Bilayer Polylactide/Cl membrane [28]. Fixation of the scaffolds was performed with titanium tack pins (TTP) [28, 29], self-drilling titanium mini-screws [31, 33] as well as resorbable pins in three studies [27, 30, 32].

In two articles, customized products were applied [30, 31] and in two [27, 29] a control group was used.

Surgical approaches were based on established guided bone regeneration principles, involving full-thickness flap elevation, placement of particulate bone graft and stabilizing scaffold. In some cases, coverage with resorbable collagen membranes was used [27, 30, 31]. Some studies employed additional techniques such as periosteal releasing incisions, while flap design and membrane fixation methods were not consistently reported. The biomaterials most frequently used were xenogeneic particulate grafts [27, 31, 32] and autogenous bone [29, 30, 33].

Perioperative medication protocols varied across studies and were inconsistently reported. Antibiotic regimens ranged from extended systemic administration for one week or longer [33] to short perioperative prophylaxis protocols (e.g., 1 day preoperatively and 2 days postoperatively in Bartols et al. [27]), while one study did not report postoperative antibiotic use [29]. The use of antiseptic agents, such as chlorhexidine rinses, was also inconsistently documented [31, 33].

Healing times before second-stage surgery also varied considerably, ranging from 3–4 months [27, 29] in cases involving less complex lateral or single-tooth defects to 7–9 months [32] in studies addressing extensive three-dimensional defects. Histologic evaluation was performed in only three studies [29–31], while the remaining investigations relied exclusively on clinical and radiographic assessments without histologic confirmation of bone maturation.

### Outcomes characteristics

The characteristics of the outcomes were described in Table 3, reporting the following items: publication details (author, year of publication), number of sites, bone gain (mm), bone volume (mm^3^), early complications, late complications, histology, remaining resorbable material, implant stability, osseointegration, follow-up (months), aesthetics (soft tissue).

Implant survival was not reported in two studies [29, 32]. In four studies, 100% survival was reported [27, 30, 31, 33]. In one study, 20% implant loss was reported [28] as reported in Table 3. Concerning primary stability, Ivanovski et al. [31] and Hsu et al. [33] reported 35Ncm and Shido et al. [29] reported equal ISQ values for all groups. The other studies did not report about primary stability of the implants. Because outcome measures for bone augmentation volume varied between studies (for example, different imaging modalities and different definitions of bone gain, units and time points), pooling of results across all studies was not straightforward. One study [28] reported differences in the area augmented (augmented volume mandibular posterior region > maxilla anterior).

Complication reporting was inconsistent across studies. Four studies [29–31, 33] had no early or late complications, out of them two were describing the utilization of customized scaffolds. Complications ranged from mucosal rubefaction and fistula [28] to exposures and bone decrease [32] and graft loss [27]. In five studies, management of complications was either not reported or not required. Management strategies ranged from local curettage and irrigation (0.2% CHX gel) in Takahara et al. [32] as well as systemic antibiosis for 4 days in Ogata et al. [28] Aesthetic outcome and appearance of soft tissue was described as “satisfying” in Schönegg et al. [30] and Ivanovski et al. [31] reported according to the Pink Esthetic Score PES 7 and WES 9 [34]. Degradation time was not mentioned in any study. Remaining particles of the scaffolds/bone augmentation process were investigated by Schönegg et al. [30]; Ivanovski et al. [31] reported about bone grafting particles.

### Meta-analysis

A quantitative meta-analysis was not performed across all outcomes because of substantial clinical and methodological heterogeneity: (1) different outcome metrics (volumetric vs. linear bone gain), (2) inconsistent reporting of dispersion measures (SD or CI) in several studies, and (3) variable follow-up and healing periods and (4) materials ((polycaprolactone, poly-D-L-lactide, poly (L-lactic acid/ε-caprolactone), PLGA, and β-TCP). This heterogeneity affects comparability and therefore, a descriptive analysis seemed to be most appropriate.

### Descriptive analysis

The included studies exhibited substantial heterogeneity in study design. One study [27] was a randomized controlled trial (RCT) conducted in Germany with 30 patients comparing autogenous bone block grafts with resorbable Poly-D-L-Lactide foil over 24 months. One prospective clinical study [29] from Japan enrolled 20 patients with 15 implant sites receiving either collagen or poly (L-lactic acid/ε-caprolactone) scaffolds with a maximum follow-up of 6 months. The remaining five studies comprised three case series [28, 32, 33] and two case reports [30, 31], with patient numbers ranging from 1 to 5 individuals and follow-up periods ranging from 5 to 48 months.

Overall, the evidence base for resorbable scaffolds is limited by moderate-to-high risk of bias in most studies, which must be taken into account when interpreting the reported clinical benefits.

### Assessment of risk of bias according higgins & altman (cochrane risk of bias domains) (Fig. 3)

**Fig. 3:**
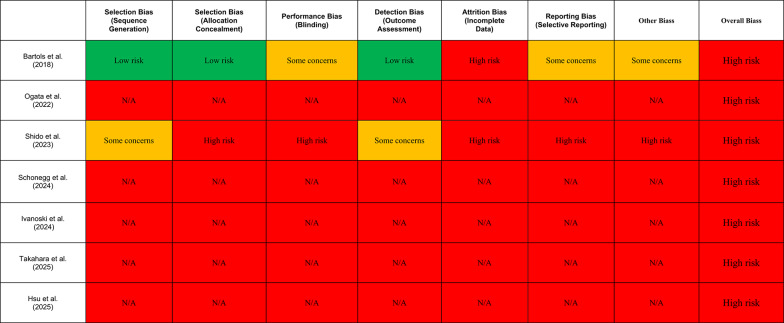
Assessment of risk of bias using the revised cochrane risk of bias tool according to higgins and altmanm. The scale is designed for randomized clinical trials

When applying the Higgins and Altman (Cochrane) risk-of-bias domains, Bartols et al. [27] clearly emerges as the only study with a methodological structure compatible with a trial-based assessment. The processes of sequence generation and, critically, allocation concealment are adequately described and therefore judged at low risk of bias, while outcome assessment benefits from standardized radiographic measurements and examiner calibration, supporting a relatively robust internal validity compared with the remaining studies. Nevertheless, a major limitation concerns incomplete outcome data, as a non-negligible proportion of radiographic outcomes could not be assessed due to graft or implant failures, resulting in informative missingness that is unlikely to be random. This issue justifies a high-risk judgment in this domain and prevents an unequivocally low overall risk-of-bias classification.

In contrast, Shido et al. [29] shows a more debatable profile. Although designed as a prospective comparative study and reporting a form of simple randomization, the methodology underlying the randomization process is insufficiently detailed, with limited information on sequence generation, allocation concealment, and operational implementation. Consequently, several key domains—particularly selection bias, performance bias, and selective reporting—must be rated as unclear or high risk. The surgical nature of the intervention and the use of different devices further complicate blinding and standardization of co-interventions, increasing susceptibility to bias due to deviations from intended interventions.

The remaining five studies [28, 30–33] demonstrate consistently poor performance under the Higgins and Altman framework (N/A, not applicable). This is primarily attributable to their observational descriptive designs (case series and case reports), which inherently lack randomization, allocation concealment, sample size, control groups, and adequate strategies to mitigate confounding and selection bias. Accordingly, these studies are characterized by an overall high risk of bias across most domains, reflecting limitations intrinsic to study design rather than deficiencies in reporting quality.

### Assessment of study quality according the newcastle–ottawa scale (NOS) (Fig. 4)

**Fig. 4:**
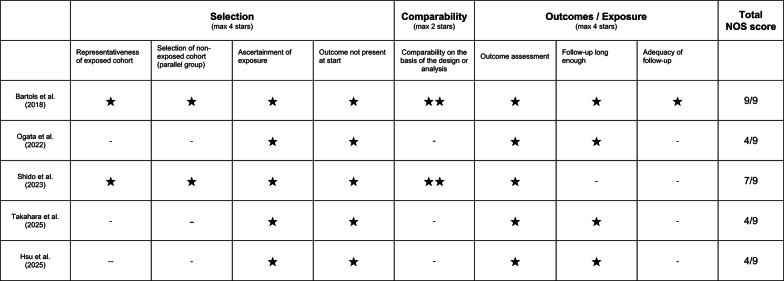
Assessment of risk of bias according to newcastle–ottawa scale (NOS). The scale is designed for non-randomized cohort studies with or without comparison. Case reports were excluded from the assessment

Application of the Newcastle–Ottawa Scale (NOS), which is specifically designed for non-randomized studies, further highlights the methodological gradient among the included articles. Shido et al. [29], interpreted as a prospective comparative cohort study, achieves an intermediate NOS score. The study performs reasonably well in the Selection and Outcome domains, owing to a clear clinical definition of the condition, standardized radiographic and clinical outcome assessment, and an appropriate follow-up period. However, the Comparability domain remains weak, as the absence of a clearly described and methodologically robust randomization process limits confidence in confounder control.

Although Bartols et al. [27] is formally outside the intended scope of NOS due to its randomized design, a conservative mapping to the NOS framework confirms its superior methodological quality relative to the remaining studies, particularly in terms of cohort selection and outcome assessment. Nonetheless, the same limitation observed in the Higgins and Altman analysis—namely the incomplete availability of outcome data due to failures—also penalizes the adequacy of follow-up item within the NOS.

The three case series [28, 32, 33] consistently yield low NOS scores, primarily because they lack any form of comparability. The absence of control groups precludes adjustment for confounding factors such as defect morphology, graft material, flap management, antibiotic regimens, timing of implant placement, and operator experience. Moreover, the small sample sizes severely limit external validity and the ability to estimate complication rates. As expected, the case reports [30, 31] cannot be meaningfully assessed in terms of representativeness or comparability.

### Assessment of study quality according joanna briggs institute (JBI) critical appraisal for case series (Fig. 5)

**Fig. 5:**
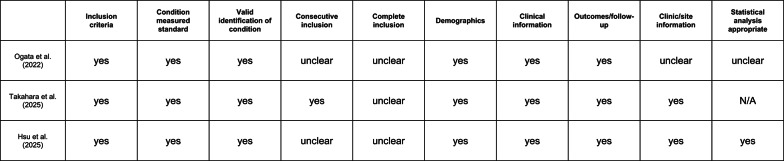
Assessment of risk of bias using the joanna briggs institute (JBI) critical appraisal tools for case series. Only case series were included in the assessment

The Joanna Briggs Institute (JBI) critical appraisal checklist for case series provides a more appropriate and nuanced assessment for descriptive studies. Ogata et al. [28], presented as a first-in-human pilot case series, demonstrates several strengths, including clearly defined inclusion criteria, detailed descriptions of the intervention, and standardized assessment of outcomes using radiographic imaging and predefined safety endpoints. However, important items remain rated as unclear, particularly whether patients were included consecutively and whether all eligible patients during the study period were enrolled, leaving the study vulnerable to selection bias.

Takahara et al. [32] performs comparatively better on several JBI items, as the recruitment appears more reflective of routine clinical practice and less exploratory than Ogata’s first-in-human design. Patient characteristics, surgical procedures, and follow-up assessments are reported in an orderly and transparent manner. Nevertheless, the inherent limitations of a small, uncontrolled case series (n = 4) persist, precluding causal inference or reliable estimation of adverse event rates.

Hsu et al. [33], despite the very limited sample size (n = 3) and a private-clinic setting, benefits from CARE-compliant reporting and a particularly rigorous radiographic methodology, including serial CBCT assessments and standardized volumetric analyses. From a JBI perspective, this results in relatively strong performance in outcome measurement and follow-up domains. However, representativeness and completeness of inclusion remain uncertain, and the lack of a comparator fundamentally limits internal validity. Overall, the JBI appraisal distinguishes differences in reporting quality among case series, while simultaneously confirming that even well-reported series remain methodologically weak for efficacy assessment.

### Assessment of study quality according joanna briggs institute (JBI) critical appraisal for case reports (Fig. 6)

**Fig. 6:**
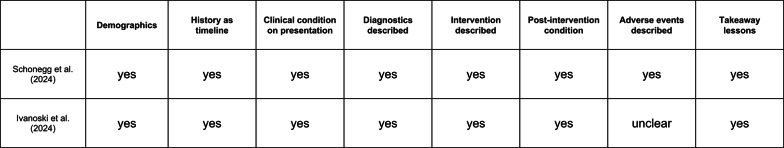
Assessment of risk of bias using the joanna briggs institute (JBI) critical appraisal tools for case reports. Only case reports were included in the assessment

For case reports, the JBI checklist focuses on completeness and transparency of clinical documentation rather than on bias control. Schönegger et al. [30] offers thorough documentation of clinical presentation, diagnostic imaging, surgical management, and long-term follow-up, supporting its value as a descriptive clinical contribution. The principal limitation lies in the reporting of adverse events, which is largely narrative (“uneventful healing”) rather than structured, potentially limiting transparency.

Similarly, Ivanoski et al. [31] performs well across most JBI items, providing clear patient demographics, a well-structured clinical timeline, detailed diagnostic work-up including CBCT imaging, comprehensive description of the intervention, and documented post-intervention outcomes. The reporting of adverse events and the inclusion of explicit clinical “take-home messages” further enhance the educational value of the report.

In both cases, the JBI appraisal confirms that these reports are well-documented and clinically informative, particularly with respect to feasibility and technical application of resorbable devices. However, their evidentiary value remains inherently limited by the absence of comparators, the high risk of publication bias, and the inability to disentangle the effect of the device from patient-specific and operator-dependent factors. As such, these studies should be interpreted as hypothesis-generating evidence rather than as support for comparative effectiveness.

## Discussion

This systematic review synthesized evidence from seven studies assessing resorbable scaffolds for bone augmentation in association with dental implant procedures. Current evidence suggests that resorbable, often patient-specific, space-maintaining frameworks (container-type scaffolds) may represent a feasible and emerging approach for alveolar ridge augmentation in the context of implant therapy; however, this is based on limited and predominantly non-comparative data.These constructs primarily function as a biological cage or barrier device, providing mechanical stability and maintaining a regenerative space. They are typically combined with additional grafting materials (e.g., particulate bone substitutes), which serve as the primary osteoconductive substrate. This is contrary to Osteoconductive scaffold matrices (block-type scaffolds).

These scaffolds act as a three-dimensional ingrowth matrix, designed to support cell migration, vascularization, and bone formation within and around the scaffold structure. In this concept, the scaffold itself provides the osteoconductive framework and may be used with or without additional grafting materials.

Across recent clinical reports and small trials, customized additive-manufactured devices composed of polycaprolactone (PCL), β-tricalcium phosphate (β-TCP), polymer-ceramic composites or bioresorbable meshes consistently provided effective space maintenance, enabled volumetric bone gain and allowed subsequent implant placement with satisfactory early stability and soft-tissue integration. Notably, recent case reports and pilot studies report volumetric bone gain following the use of PCL-based, patient-specific scaffolds in complex three-dimensional defects [28, 29, 31, 33].

A potential advantage of additively manufactured resorbable scaffolds may be the structural “cage effect”: an accurately contoured scaffold stabilizes particulate graft material (in the provided studies mostly xenogeneic and autogenous particles), resists soft-tissue collapse and creates a protected osteoconductive chamber that favors vascular infiltration and bone formation. Preclinical and translational studies like Vaquette et al.[35] support this concept and demonstrate that pore architecture, interconnectivity and material composition (e.g., PCL–hydroxyapatite blends) influence early cell ingrowth, angiogenesis and mineralization kinetics.

However, the evidence base remains limited by considerable heterogeneity in defect morphology, scaffold materials, adjunctive grafts and surgical technique like shown in this review. Recent randomized and non-randomized clinical investigations have used diverse interventions—from customized ceramic (3D-printed β-TCP) grafts in a randomized setting to PCL scaffolds combined with biologics such as platelet-rich fibrin (PRF) or BMP-loaded hydrogels—complicating direct comparisons [36]. For example, a recent randomized clinical trial evaluating customised 3D-printed ceramic grafts reported favourable volumetric outcomes but used different fixation and adjunct protocols than contemporary PCL case series, thus limiting head-to-head inference [37].

A recurrent concern across multiple reports is scaffold degradation kinetics [30, 31, 38–40]. Several clinical case series and pilot trials documented residual scaffold material at intermediate follow-up, indicating slower resorption than anticipated. While overt foreign-body reactions were not reported in the available clinical material, the long-term biological consequence of persistent polymer fragments—including potential low-grade inflammation or mechanical interference with late remodeling and implant load transfer—remains insufficiently characterized and requires targeted long-term surveillance[41]. The lack of data evaluated in this systematic review represents a significant gap, as degradation behaviour is a key factor influencing the long-term clinical performance and predictability of the investigated materials. Future studies should therefore systematically assess and report degradation time to enable a more comprehensive evaluation of clinical outcomes.

Complication reporting in the current literature is inconsistent. Most studies of this systematic review described none; followed by infrequent and predominantly minor complications (small membrane exposures, transient mucosal irritation, superficial dehiscence)[32, 33]; just few studies described partial or total graft loss in subsets of cases [27]. Notably, the definition and classification of complications varied considerably across studies, ranging from exposures and infections to bone graft loss. Therefore, all reported complication types were described and considered in this review to ensure comprehensive and transparent reporting. Recent innovations such as bioresorbable mesh domes (Vicryl-based) demonstrate feasible handling and favorable early outcomes in small series, but their clinical safety profile requires larger cohorts and standardized adverse-event documentation [42]. Across included studies, differences in perioperative antibiotic/antiseptic regimes and flap management (including periosteal releasing incisions and flap designs) further confound complication rates and healing outcomes.

Methodological limitations persist. The majority of available clinical evidence consists of case reports, case series, or small pilot randomized trials with limited statistical power and variable follow-up durations. Histological endpoints are inconsistently reported and are often opportunistic (e.g., biopsies obtained at implant placement), while volumetric assessments vary in methodology (e.g., differing CBCT segmentation protocols). Few studies were preregistered or reported sample size calculations, and blinding of outcome assessment was infrequent. These limitations increase the risk of selection and detection bias and limit external validity [39, 43].

Based on the currently available evidence, resorbable customized scaffolds may represent a potential option for complex three-dimensional alveolar reconstruction, particularly in situations where avoidance of donor-site morbidity and elimination of second-stage mesh removal are desired. However, given the limited and heterogeneous data, these findings should be interpreted with caution.

Material selection may need to be individualized based on factors such as defect size and containment, the anticipated requirement for space maintenance, the degradation characteristics of the scaffold, available fixation options, and the clinician’s experience with the respective device. However, these considerations are derived from limited and heterogeneous evidence and should therefore be interpreted as practice-oriented observations rather than evidence-based recommendations [44].

Future research should aim to generate evidence with adequately powered randomized controlled trials comparing defined resorbable scaffold systems with established augmentation methods (titanium meshes, autogenous block grafts, split bone bloque techniques [13, 45]). Clinical trials should adopt volumetric CBCT protocols, standard histomorphometric endpoints (including vital bone %, residual scaffold %), prespecified complication reporting and managing protocols, and include patient-reported outcomes and cost-effectiveness analyses. Translational work should clarify degradation products, host immunologic responses and long-term mechanical integration under functional loading.

Despite addressing a highly innovative and clinically relevant topic, this systematic review is constrained by the low level and heterogeneity of the available evidence. Only seven studies met the inclusion criteria, mostly case reports or small case series, with a single randomized controlled trial and one prospective comparative study, resulting in limited sample sizes and low statistical power. The overall risk of bias was high in most studies, and even the RCT presented concerns related to incomplete outcome data. Marked clinical and methodological heterogeneity—regarding defect morphology, surgical protocols, grafting materials, timing of implant placement, and outcome assessment—precluded quantitative synthesis and meta-analysis. Moreover, key aspects directly related to the novelty of resorbable barriers, such as degradation kinetics and long-term behavior of the devices, were poorly reported or entirely absent. Histological data and long-term follow-up were available only in a minority of studies, further limiting the strength of the conclusions.

Despite these limitations, this review provides a structured synthesis of the available evidence, including detailed reporting of surgical techniques, biomaterials, and outcomes, which supports cautious clinical interpretation. The findings suggest early indications of feasibility and acceptable safety of resorbable, including patient-specific and 3D-printed, barrier systems; however, these observations are based on limited and predominantly non-comparative data. Overall, resorbable approaches should be considered investigational and require validation in well-designed randomized clinical trials. In conclusion, the addition of recent clinical and translational studies strengthens the overall conclusion of this review: the concept is fundamentally interesting and may offer potential advantages over conventional techniques, but performance must still be demonstrated in high-quality studies.

Nevertheless, heterogeneity of materials and methods, limited high-quality comparative data and unanswered questions about long-term scaffold behaviour mandate cautious clinical adoption and underscore the need for rigorous randomized comparative trials with standardised endpoints.

## Conclusion

The included studies collectively indicate that resorbable scaffolds can support bone regeneration and enable implant placement in a range of augmentation procedures, with generally acceptable safety profiles. Nevertheless, heterogeneity in materials, surgical protocols, and outcome reporting—together with methodological limitations and mainly short-to-intermediate follow-up in recent trials—prevents definitive conclusions about the relative superiority of particular scaffold types. Standardized, longer-term randomized trials are needed to establish optimal scaffold selection and to confirm the durability of the clinical benefits observed in early studies. Degradation time is a key determinant of long-term clinical performance and warrants systematic investigation in future research.

## Data Availability

The data that support the findings of this study are available from the corresponding author upon reasonable request.
